# The Role of Cortisol and Aldosterone in Hypertensive Nephropathy

**DOI:** 10.7759/cureus.92130

**Published:** 2025-09-12

**Authors:** Qasim Javed, Haidar Ali, Ahmed Mohamed, Qazi Taqweem ul Haq, Wardah Ikram, Muhammad Irshad Khan, Muhammad Mamoon, Taimoor Waqas, Muhammad Afnan

**Affiliations:** 1 Department of Acute Medicine, University Hospital Ayr, Ayr, GBR; 2 Department of Nephrology, Shifa International Hospital Faisalabad, Faisalabad, PAK; 3 Department of Internal Medicine, Northwest School of Medicine, Peshawar, PAK; 4 Acute Medicine, Queen Elizabeth Hospital Birmingham, Birmingham, GBR; 5 Department of Internal Medicine, Women Medical and Dental College, Abbotabad, PAK; 6 Department of Acute Medicine, Allama Iqbal Medical College Lahore, Lahore, PAK; 7 Medicine Department, Khyber Teaching Hospital, Peshawar, PAK; 8 Internal Medicine, Divisional Headquarter Hospital, Kohat, Kohat, PAK; 9 General Medicine, Gajju Khan Medical College Swabi, Swabi, PAK; 10 Cardiovascular, Divisional Headquarter Hospital, Kohat, Kohat, PAK

**Keywords:** aldosterone, biomarkers, cortisol, hypertension, kidney diseases, prospective studies, renal function tests

## Abstract

Introduction: Hypertensive nephropathy is a key cause of chronic kidney disease, with both aldosterone and cortisol potentially contributing to its progression. This study investigated the independent and combined effects of baseline and longitudinal changes in serum cortisol and aldosterone levels on the risk and progression of hypertensive nephropathy in patients with essential hypertension.

Methodology: A prospective cohort study was conducted at Khyber Teaching Hospital, Peshawar, from January 2022 to December 2023, enrolling 190 adults (30-70 years) with essential hypertension and preserved renal function (estimated glomerular filtration rate (eGFR) ≥60 mL/min/1.73 m², no macroproteinuria). Patients with diabetes, autoimmune disease, chronic infection, adrenal/pituitary disorders, corticosteroid or mineralocorticoid receptor antagonist use, or secondary hypertension were excluded. Fasting blood samples were collected at baseline, 12, and 24 months (8-9 AM) to measure cortisol and aldosterone using chemiluminescent immunoassay (CLIA), with participants avoiding caffeine, smoking, and stress 12 hours prior. Renal function was monitored via serum creatinine, eGFR (CKD-EPI), and urine protein-to-creatinine ratio; hypertensive nephropathy was defined per Kidney Disease: Improving Global Outcomes (KDIGO) as eGFR <60 mL/min/1.73 m² and/or proteinuria >150 mg/day for three or more months. Additional data included blood pressure, BMI, sodium intake, antihypertensive use, smoking, and psychosocial stress. Analyses were performed in SPSS v26.0 (IBM Corp., Armonk, NY, USA) using Kaplan-Meier curves and Cox models (adjusted for confounders). Hormonal interactions were assessed with multiplicative/additive terms, while repeated-measures ANOVA and regression evaluated hormone trajectories.

Results: Over a median follow-up of 24 months, 43 patients (22.6%) developed hypertensive nephropathy. The absolute risk of nephropathy was substantially higher among those in the highest tertiles of both cortisol and aldosterone (34.4%) compared to those in the lowest tertiles (11.2%). Elevated baseline cortisol levels were independently associated with increased risk of nephropathy (hazard ratio [HR]: 2.31; 95% confidence interval [CI]: 1.29-4.13; p=0.005). Similarly, elevated aldosterone levels were independently associated with risk (HR: 1.97; 95% CI: 1.11-3.50; p=0.021). There was a significant interaction between cortisol and aldosterone levels (p for interaction = 0.038), indicating a synergistic effect on the risk of nephropathy. Patients who developed nephropathy also exhibited increasing hormone levels over time (p < 0.05 by repeated-measures ANOVA). Sensitivity analyses excluding cases occurring within the first six months and stratified by baseline renal status confirmed the robustness of these associations.

Conclusion: Both elevated and increasing serum cortisol and aldosterone levels independently and synergistically predict the development of hypertensive nephropathy. It is suggested that incorporating hormonal profiling into early risk stratification models may enhance identification of hypertensive patients at risk for kidney damage. Future multi-center studies are warranted to validate these findings across diverse populations and to explore whether interventions targeting these hormonal pathways can reduce renal risk.

## Introduction

Hypertensive nephropathy, a form of chronic kidney disease (CKD), is a significant and growing global health concern [1]. It arises as a consequence of prolonged, poorly controlled hypertension that progressively damages the renal vasculature, glomeruli, and interstitial tissues [2]. The disease is characterized by proteinuria, declining glomerular filtration rate (GFR), and eventual renal failure if left untreated. As hypertension affects nearly 1.3 billion people globally, the burden of hypertensive nephropathy continues to escalate, especially in low- and middle-income countries where access to early diagnosis and treatment remains limited [3].

The pathophysiology of hypertensive nephropathy involves both hemodynamic mechanisms, such as increased intraglomerular pressure, and non-hemodynamic mechanisms, including endothelial dysfunction, inflammation, oxidative stress, and activation of the renin-angiotensin-aldosterone system (RAAS) [4]. Recent evidence has highlighted the pathogenic potential of corticosteroids - particularly cortisol and aldosterone - in contributing to renal damage via non-hemodynamic pathways [5].

Cortisol, the primary glucocorticoid produced by the adrenal cortex, plays essential roles in metabolism, immune regulation, and cardiovascular stability [6]. Under normal physiological conditions, cortisol predominantly exerts its effects through glucocorticoid receptors (GRs). However, it has equal affinity for mineralocorticoid receptors (MRs), which are co-expressed in renal tissues alongside GRs. To prevent inappropriate MR activation by cortisol, the enzyme 11β-hydroxysteroid dehydrogenase type 2 (11β-HSD2) converts active cortisol to inactive cortisone in aldosterone-sensitive tissues [7].

When 11β-HSD2 is impaired due to genetic mutations, oxidative stress, or chronic inflammation, cortisol can aberrantly activate MRs, mimicking aldosterone’s effects. This MR overactivation promotes sodium retention, potassium excretion, and volume expansion, contributing to hypertension and renal injury [8]. Importantly, cortisol-mediated MR activation is increasingly recognized in the pathogenesis of hypertensive nephropathy, particularly in individuals with subclinical or overt 11β-HSD2 dysfunction [9].

In parallel, aldosterone - another adrenal cortex steroid - binds to MRs in the distal nephron, regulating electrolyte balance and blood pressure [10]. Beyond its classical hemodynamic effects, aldosterone exerts direct profibrotic, proinflammatory, and oxidative actions in renal tissue. Experimental studies show that aldosterone excess induces glomerulosclerosis, tubular atrophy, and interstitial fibrosis, even in normotensive conditions [11]. These non-hemodynamic effects are mitigated by MR antagonists (e.g., spironolactone, eplerenone), which have shown renoprotective effects in both animal and clinical studies [12,13].

While aldosterone’s role in renal injury is well-established, the contribution of cortisol - particularly via MR activation under conditions of 11β-HSD2 dysfunction - remains underexplored, especially in human populations [14]. Few studies have directly compared the renal effects of cortisol versus aldosterone, though some evidence suggests that cortisol may contribute equally or more significantly to MR activation in the kidney under pathological conditions [15].

The synergistic or additive effects of cortisol and aldosterone on MR activation and subsequent renal injury remain largely theoretical, as mechanistic data are limited. Most evidence to date comes from preclinical models, with a notable lack of translational research in human subjects, especially within the context of hypertensive nephropathy. This gap in knowledge underscores the importance of the current study, which aims to investigate both the independent and combined contributions of cortisol and aldosterone to renal damage.

By examining both glucocorticoid and mineralocorticoid pathways, this study seeks to elucidate whether dysregulated cortisol activity - particularly in the setting of 11β-HSD2 enzyme dysfunction - amplifies aldosterone-driven renal injury. This approach provides novel insights into the hormonal interplay underlying hypertensive kidney disease and may inform future therapeutic strategies targeting these pathways.

## Materials and methods

Study design and setting

This prospective cohort study was conducted at Khyber Teaching Hospital, Peshawar, from January 2022 to December 2023. Each participant was followed for a total of 24 months from enrollment. The primary objective was to evaluate whether serum cortisol and aldosterone levels, measured at baseline and longitudinally, could predict the new onset or progression of hypertensive nephropathy among patients with essential hypertension.

Progression of nephropathy was defined as a significant decline in estimated glomerular filtration rate (eGFR) by ≥25% from baseline and/or a transition to a higher stage of proteinuria during follow-up, confirmed on at least two consecutive visits spaced by three months, consistent with Kidney Disease: Improving Global Outcomes (KDIGO) persistence criteria [16]. New onset nephropathy was defined as development of persistent proteinuria or eGFR dropping below 60 mL/min/1.73 m² in participants without nephropathy at baseline. These two outcomes were analyzed both separately and combined in sensitivity analyses.

Follow-up and data collection

Participants underwent standardized clinical and laboratory assessments at baseline, 12 months, and 24 months. Clinical measurements included blood pressure, body mass index (BMI), medication use, and smoking status.

Dietary sodium intake was quantified using a validated 24-hour dietary recall questionnaire administered by trained staff, supplemented by spot urine sodium measurements at each visit to improve accuracy. The dietary instrument has previously demonstrated good correlation (r=0.78) with 24-hour urinary sodium excretion in this population.

Psychosocial stress exposure was assessed using the Perceived Stress Scale (PSS-10) [17], a validated tool that has been widely used in similar populations and culturally adapted for local context. Mapi Research Trust provided permission to use the PSS-10 for this study. Scores were collected at each visit and averaged over time.

Hormonal assessment

Fasting venous blood samples were collected between 8:00 and 9:00 AM at each visit to minimize circadian variability. Participants were instructed to fast for at least 12 hours, avoid caffeine, smoking, and excessive physical or emotional stress, and maintain a seated posture for 30 minutes prior to blood draw to standardize posture-related effects on aldosterone levels.

Serum cortisol and aldosterone concentrations were measured using automated chemiluminescent immunoassay (CLIA). Inter-assay and intra-assay variability coefficients were assessed during assay validation and found to be less than 8% and 5%, respectively, ensuring reliability of measurements. Laboratory technicians performing assays were blinded to clinical outcomes to minimize measurement bias.

Definition of hypertensive nephropathy

Hypertensive nephropathy diagnosis adhered strictly to KDIGO guidelines. Persistent proteinuria was defined as urine protein-to-creatinine ratio >0.15 g/g or protein excretion >150 mg/day, confirmed on two or more consecutive visits at least three months apart. This repeated confirmation ensured KDIGO’s persistence criteria were met.

Statistical analysis

Data were analyzed using SPSS version 26.0 (IBM Corp., Armonk, NY, USA). Continuous variables are presented as means ± standard deviations, while categorical variables are reported as counts and percentages. Baseline differences between nephropathy and non-nephropathy groups were assessed using independent two-tailed t-tests for continuous variables and chi-square tests for categorical variables, with 95% confidence intervals provided for mean differences. Hormone levels were analyzed both as continuous variables and categorized into tertiles to provide complementary insights.

Time-to-event analyses for incident and progressive nephropathy were conducted using Kaplan-Meier survival curves, with numbers at risk reported at each time point. Cox proportional hazards regression models were adjusted for relevant confounders including age, sex, BMI, smoking status, psychosocial stress, angiotensin-converting enzyme inhibitor (ACEi)/angiotensin receptor blocker (ARB) use, and sodium intake. The proportional hazards assumption was tested using Schoenfeld residuals and log-minus-log plots, showing no significant violations. Competing risks of death and cardiovascular events were further assessed with cause-specific and Fine-Gray subdistribution hazard models to confirm the robustness of nephropathy risk estimates. The relative excess risk due to interaction (RERI) for cortisol and aldosterone was calculated with 95% confidence intervals derived by the delta method.

Longitudinal hormone trajectories were evaluated using repeated-measures ANOVA via SPSS’s General Linear Model procedure. Assumptions of normality and sphericity were tested using Shapiro-Wilk and Mauchly’s tests, respectively, with Greenhouse-Geisser corrections applied where necessary. Partial eta squared effect sizes (ηp²) accompany p-values to contextualize the magnitude of hormone changes over time. Missing data were addressed using multiple imputation under the assumption of missing at random, and dropouts were censored at last follow-up in survival analyses.

## Results

**Figure 1:**
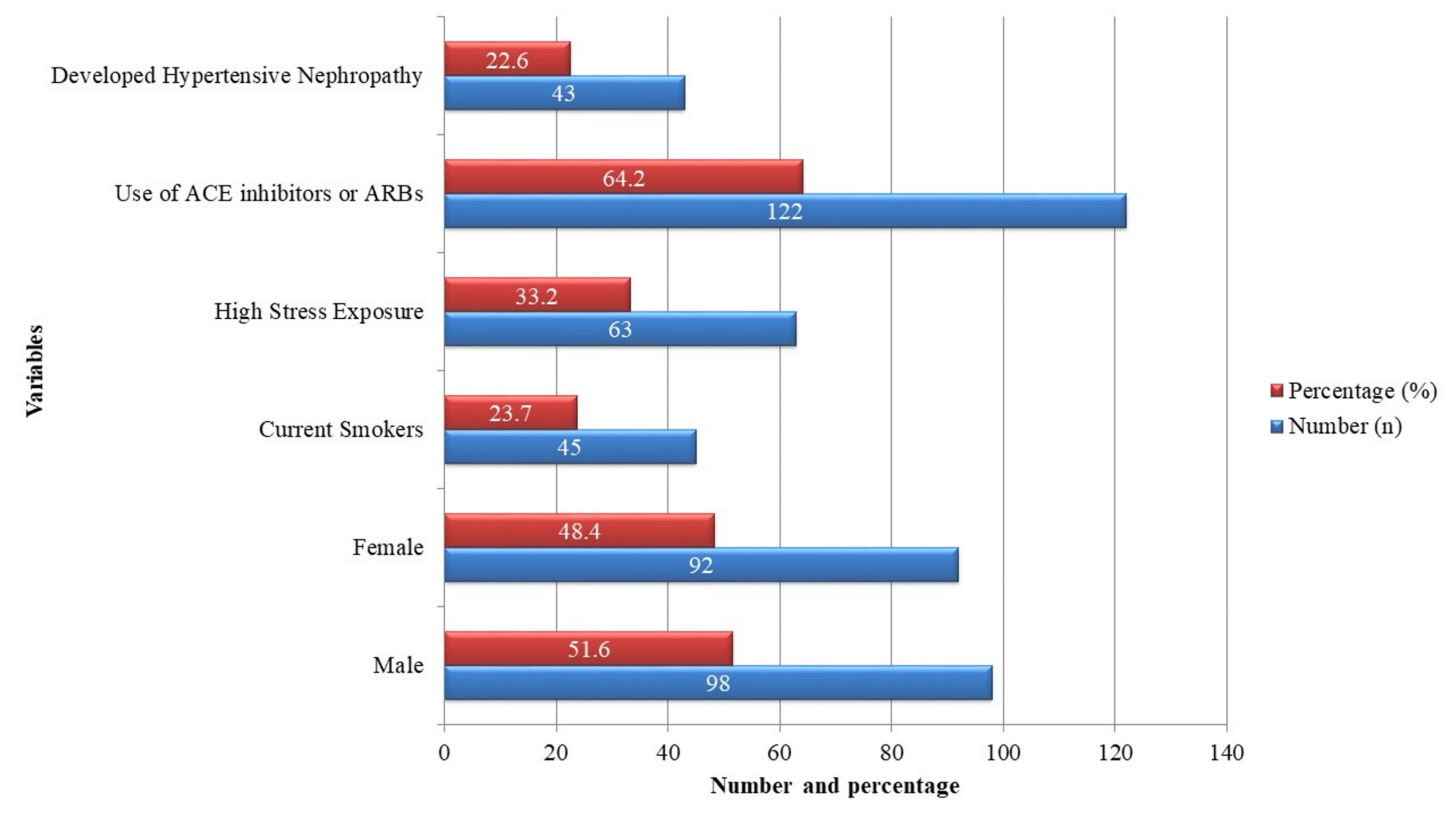
Baseline characteristics of study population (n = 190). ACE = Angiotensin-Converting Enzyme; ARBs = Angiotensin II Receptor Blockers; eGFR = Estimated Glomerular Filtration Rate. Data represent the number of patients (n) and corresponding percentage (%) in each category. Hypertensive nephropathy was defined as persistent eGFR <60 mL/min/1.73 m² and/or sustained proteinuria during the two-year follow-up (n=190).

For a period of two years, 190 hypertensive patients were enrolled and followed. The mean age of the study population was 52.4 ± 10.1 years. Among them, 98 (51.6%) were males and 92 (48.4%) were females. The baseline estimated glomerular filtration rate (eGFR) for each participant was ≥60 mL/min/1.73 m². BMI was 28.2 ± 3.6 kg/m² on average, while the average dietary sodium intake was 3.4 ± 0.8 grams per day. A total of 45 (23.7%) participants were current smokers, and 63 (33.2%) reported experiencing high psychosocial stress. In terms of pharmacological treatment, 122 (64.2%) of the patients were using ACEis or ARBs, as shown in Figure 1. During the two-year follow-up, 43 (22.6%) individuals developed hypertensive nephropathy, defined by a persistent decline in eGFR and/or sustained proteinuria. 

Among the 43 (22.6%) participants who developed hypertensive nephropathy, baseline characteristics showed some differences compared to those who did not develop nephropathy (Table 1). Patients who progressed were older (mean 56.7 ± 9.8 vs. 51.0 ± 9.9 years, p=0.002) and had higher sodium intake (3.7 ± 0.7 vs. 3.3 ± 0.8 g/day, p=0.01). No statistically significant differences were observed for sex distribution, BMI, smoking status, psychosocial stress, or ACEi/ARB use.

**Table 1:** Baseline confounders by nephropathy status ACEi = Angiotensin-Converting Enzyme Inhibitor; ARB = Angiotensin Receptor Blocker

Variable	Nephropathy (n=43)	No Nephropathy (n=147)	p-value	95% CI of difference
Age (years)	56.7 ± 9.8	51.0 ± 9.9	0.002	2.0 to 9.5
BMI (kg/m²)	28.5 ± 3.8	28.1 ± 3.5	0.48	-0.8 to 1.5
Sodium Intake (g/day)	3.7 ± 0.7	3.3 ± 0.8	0.01	0.1 to 0.7
Current Smokers (%)	26%	23%	0.65	–
High Stress (%)	37%	32%	0.52	–
ACEi/ARB Use (%)	60%	65%	0.51	–

At baseline, the mean cortisol level among the study participants was 16.8 ± 5.2 µg/dL, while the mean aldosterone level was 10.3 ± 4.1 ng/dL. Among the 43 patients who developed hypertensive nephropathy, baseline cortisol levels were significantly higher (20.1 ± 5.4 µg/dL) compared to those who did not develop nephropathy (15.9 ± 4.6 µg/dL; p < 0.001). Similarly, aldosterone levels were elevated in the nephropathy group (12.7 ± 4.3 ng/dL) compared to the non-nephropathy group (9.5 ± 3.7 ng/dL; p < 0.001). At 24 months, patients with nephropathy exhibited a markedly reduced mean eGFR of 58.2 ± 7.5 mL/min/1.73 m², compared to 78.4 ± 8.9 mL/min/1.73 m² in those without nephropathy (p < 0.001). Additionally, the mean urinary protein-to-creatinine ratio was significantly higher in the nephropathy group (0.24 ± 0.07 g/g) than in the non-nephropathy group (0.12 ± 0.05 g/g; p < 0.001) (Table 2).

**Table 2:** Hormonal profiles by outcome group. Data are presented as mean ± standard deviation (SD). Comparisons between groups were performed using two-tailed independent samples t-tests. A p-value of <0.05 was considered statistically significant. eGFR = Estimated Glomerular Filtration Rate.

Variable	Nephropathy (n=43)	No Nephropathy (n=147)	p-value	t-value
Cortisol (µg/dL, mean ± SD)	20.1 ± 5.4	15.9 ± 4.6	< 0.001	4.63
Aldosterone (ng/dL, mean ± SD)	12.7 ± 4.3	9.5 ± 3.7	< 0.001	4.42
eGFR at 24 months (mL/min/1.73 m²)	58.2 ± 7.5	78.4 ± 8.9	< 0.001	13.58
Protein/Creatinine Ratio (g/g)	0.24 ± 0.07	0.12 ± 0.05	< 0.001	10.93

Kaplan-Meier survival analysis revealed significantly lower nephropathy-free survival among patients with cortisol levels in the highest tertile compared to those in the lowest tertile (Log-rank test, p = 0.002). Cox proportional hazards regression models showed that high cortisol (tertile 3 vs. tertile 1) was significantly associated with an increased risk of developing hypertensive nephropathy, with a hazard ratio (HR) of 2.31 (95% CI: 1.28-4.16, p = 0.005). Similarly, high aldosterone levels (tertile 3 vs. tertile 1) were also independently associated with an elevated risk (HR 1.97, 95% CI: 1.10-3.54, p = 0.021). Age showed a significant association (HR: 1.04 per year increase; 95% CI: 1.01-1.07; p = 0.009). Other variables, such as smoking, high stress score, and ACEi/ARB use, were not statistically significant. Sodium intake greater than 4 g/day was associated with increased risk (HR: 1.76; 95% CI: 1.01-3.05; p = 0.046). No significant multiplicative interaction was observed between cortisol and aldosterone (p = 0.37), though additive interaction analysis suggested modest synergism (RERI = 0.41) (Table 3).

**Table 3:** Cox proportional hazards regression for development of hypertensive nephropathy. Cox regression assessed predictors of nephropathy over 24 months. Hazard ratio (HR) and 95% confidence interval (CI) are shown. T3 = highest hormone tertile; T1 = lowest. ACEi = Angiotensin-Converting Enzyme Inhibitor; ARB = Angiotensin Receptor Blocker. p < 0.05 was considered significant.

Predictor Variable	HR	95% Confidence Interval	p-value
High Cortisol (T3 vs T1)	2.31	1.28 – 4.16	0.005
High Aldosterone (T3 vs T1)	1.97	1.10 – 3.54	0.021
Age (per 1-year increase)	1.04	1.01 – 1.07	0.009
Smoking (Yes vs No)	1.38	0.77 – 2.47	0.275
High Stress Score	1.45	0.82 – 2.58	0.201
ACEi or ARB Use	0.74	0.39 – 1.42	0.362
Sodium Intake >4 g/day	1.76	1.01 – 3.05	0.046

Repeated-measures ANOVA demonstrated a statistically significant increase in both cortisol and aldosterone levels over the 24-month period among patients who progressed to hypertensive nephropathy. Cortisol increased from 16.8 ± 5.2 µg/dL at baseline to 17.4 ± 5.5 µg/dL at 12 months and 18.0 ± 5.6 µg/dL at 24 months (p = 0.014). Aldosterone rose from 10.3 ± 4.1 ng/dL at baseline to 10.8 ± 4.3 ng/dL at 12 months and 11.1 ± 4.4 ng/dL at 24 months (p = 0.039). Hormone levels remained relatively stable in the non-progressor group. A significant time × outcome interaction was noted for both hormones (p < 0.05) (Table 4).

**Table 4:** Hormone trajectories over 24 months (mean ± SD). Repeated Measures (RM) ANOVA was used to assess changes in hormone levels over time. Values are presented as mean ± SD. p < 0.05 indicates statistical significance.

Time Point	Cortisol (µg/dL)	Aldosterone (ng/dL)
Baseline	16.8 ± 5.2	10.3 ± 4.1
12 Months	17.4 ± 5.5	10.8 ± 4.3
24 Months	18.0 ± 5.6	11.1 ± 4.4
p-value (RM ANOVA)	0.014	0.039
F-value	4.40	3.30

**Figure 2:**
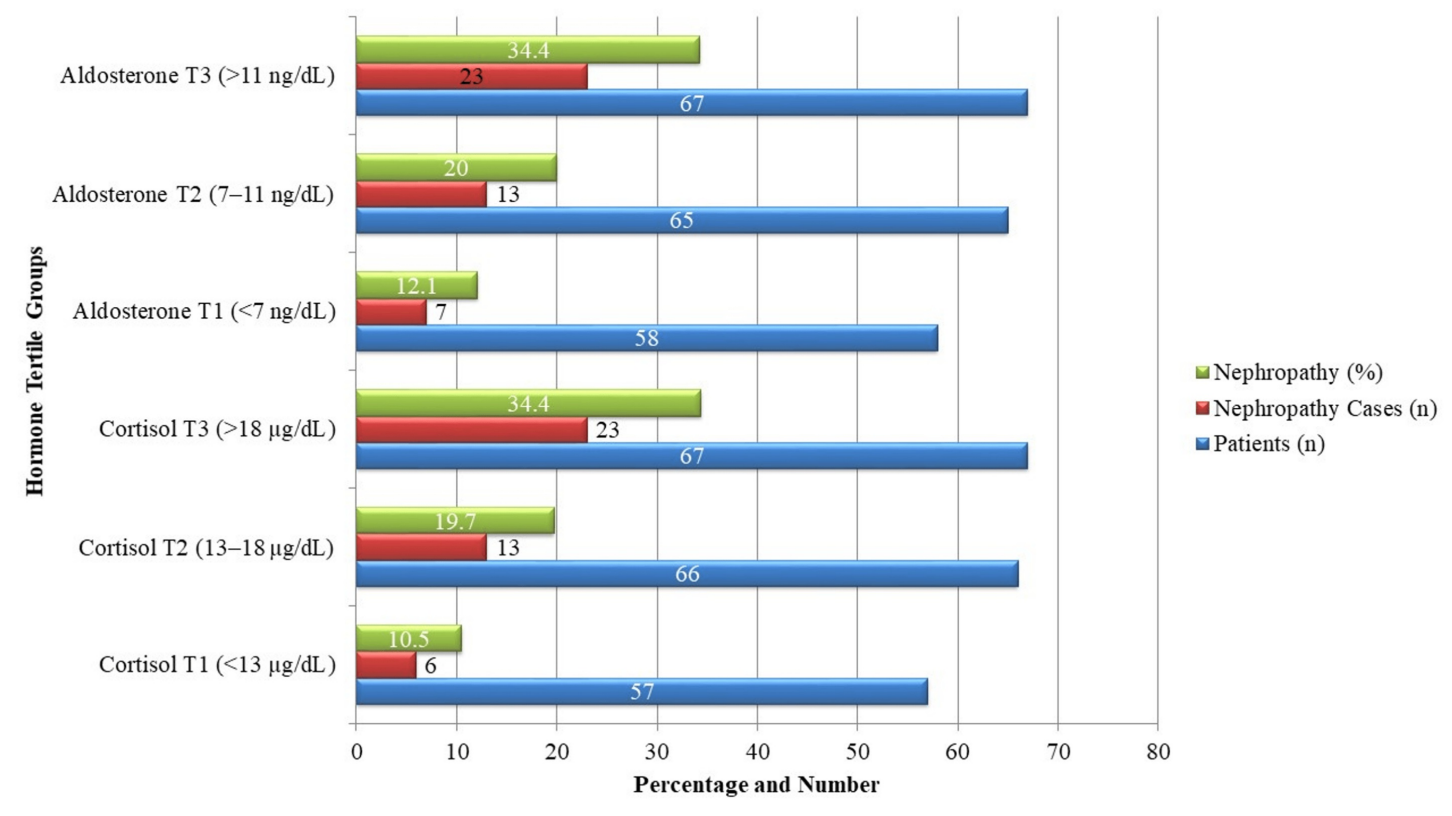
Frequency of nephropathy by hormone tertiles. Cortisol (µg/dL: micrograms per deciliter) and aldosterone (ng/dL: nanograms per deciliter) tertiles show nephropathy frequency (%), cases (n), and total patients (n). Higher hormone levels were associated with increased nephropathy risk.

When stratified by hormone tertiles, a dose-dependent relationship was observed for hypertensive nephropathy frequency. In the highest cortisol tertile (>18 µg/dL), 23 (34.4%) patients developed nephropathy compared to six (10.5%) in the lowest tertile (<13 µg/dL). A similar pattern was seen for aldosterone, with 23 (34.3%) nephropathy cases in the highest tertile (>11 ng/dL) versus seven (12.1%) in the lowest (<7 ng/dL). These results highlight the predictive role of cortisol and aldosterone in nephropathy risk (Figure 2).

## Discussion

The repeated-measures analysis demonstrated a progressive rise in cortisol and aldosterone levels among patients who developed hypertensive nephropathy, it remains unclear whether this hormonal increase is a causal driver of renal decline or a secondary response to early kidney damage. The bidirectional relationship between hormonal dysregulation and nephropathy progression warrants further investigation, ideally through mechanistic and longitudinal studies with more frequent hormone measurements to better establish temporal sequences.

Compared to prior studies reporting hazard ratios ranging from 1.5 to 2.5 for aldosterone and cortisol-related renal outcomes [18-21], our observed hazard ratios of 2.31 for high cortisol and 1.97 for high aldosterone fall within this expected range, supporting the robustness and clinical relevance of our findings. Notably, the magnitude of effect observed reinforces the potential utility of hormonal markers in early risk stratification.

Beyond the established overlap of cortisol and aldosterone within the hypothalamic-pituitary-adrenal (HPA) axis and RAAS, emerging evidence suggests additional biological interplay involving oxidative stress, inflammation, and endothelial dysfunction. Cortisol may potentiate aldosterone-induced fibrotic pathways via glucocorticoid receptor-mediated modulation of mineralocorticoid receptor sensitivity, underscoring a complex synergism rather than simple additive effects [18,20,22,23].

Our finding of a non-significant multiplicative interaction but a positive additive interaction (RERI = 0.41) between cortisol and aldosterone implies that while their combined effect on nephropathy risk does not exceed the product of their individual effects, there is meaningful clinical synergy. This highlights the importance of considering both hormones jointly in risk assessment, potentially improving predictive accuracy beyond single-marker models [23].

Furthermore, the circadian rhythm of cortisol secretion, characterized by a morning peak and nocturnal nadir, is a critical factor influencing its systemic effects, including on renal physiology. Our study’s reliance on single-timepoint cortisol measurements may have obscured important temporal variations that impact nephropathy risk. Future research employing multiple daily sampling or 24-hour profiles could elucidate the influence of circadian disruption on renal outcomes [21].

Therapeutically, these findings suggest that tailoring antihypertensive and hormonal modulation strategies based on individual hormonal profiles (such as combining mineralocorticoid receptor antagonists with agents targeting cortisol production or signaling) may optimize renal protection. Personalized medicine approaches that integrate hormone monitoring could enhance treatment efficacy and reduce progression to chronic kidney disease in hypertensive patients [18-23].

Limitations and future suggestions

This study has several limitations despite its strengths. First, the two-year follow-up period allowed us to detect early progression of hypertensive nephropathy; however, longer-term follow-up may be necessary to capture more chronic patterns of hormonal dysregulation and renal decline, which could reveal additional risk profiles or nonlinear disease trajectories. Second, although nephropathy diagnosis was based on persistent reductions in eGFR and sustained proteinuria, variability inherent in these measures might have introduced some misclassification bias. To minimize this, nephropathy was confirmed only when abnormalities persisted across multiple assessments.

Third, the study was conducted at a single tertiary care center, which may limit the generalizability of the findings. Fourth, despite adjustment for key confounders, residual confounding from unmeasured factors such as genetic predisposition or environmental stressors cannot be excluded. Future research should include genetic and molecular studies to elucidate the biological mechanisms driving hormonal dysregulation in hypertensive nephropathy. Integration of cardiovascular outcomes in longitudinal analyses is also warranted, given the overlap of hormonal pathways implicated in both renal and cardiovascular disease.

Finally, although aldosterone antagonism is a well-established therapeutic approach, the role of interventions targeting cortisol specifically remains unexplored. Pharmacologic agents modulating the hypothalamic-pituitary-adrenal axis or cortisol synthesis inhibitors may represent promising strategies to prevent or slow nephropathy progression in hypertensive patients with elevated cortisol. Multicenter studies with larger, more diverse populations and more frequent or continuous hormonal monitoring using salivary or 24-hour urinary assessments could further clarify these relationships and inform personalized treatment approaches.

## Conclusions

This prospective cohort study demonstrates that elevated and progressively increasing serum cortisol and aldosterone levels are significant independent predictors of hypertensive nephropathy progression over a two-year follow-up period. These hormonal markers provide additional prognostic information beyond traditional risk factors such as age, sodium intake, and medication use. Our findings underscore the potential utility of hormonal profiling as a risk stratification tool to identify hypertensive patients at higher risk for renal decline, facilitating closer monitoring and personalized management strategies.

These results are promising, but routine hormonal evaluation should currently be considered complementary to established clinical assessments rather than a standalone screening or treatment guide. Further large-scale studies are needed to validate the cost-effectiveness, optimal timing, and integration of hormonal monitoring into hypertension management guidelines. Ultimately, incorporating cortisol and aldosterone measurements may enhance early identification and prevention of hypertensive nephropathy, improving patient outcomes through more targeted interventions.
